# Ganglioglioma of the Right Cerebrothalamus in a 7-Year-Old Quarter Horse Cross Gelding

**DOI:** 10.3389/fvets.2019.00356

**Published:** 2019-10-22

**Authors:** Charlotte Easton-Jones, Kevin Woolard, F. Charles Mohr, Melissa A. Roy, Monica Aleman

**Affiliations:** ^1^School of Veterinary Medicine, The William R. Pritchard Veterinary Medical Teaching Hospital, University of California, Davis, Davis, CA, United States; ^2^Department of Pathology, Microbiology, and Immunology, School of Veterinary Medicine, University of California, Davis, Davis, CA, United States; ^3^Department of Medicine and Epidemiology, School of Veterinary Medicine, University of California, Davis, Davis, CA, United States

**Keywords:** horse, brain, electroencephalography, glial, neoplasia

## Abstract

Intracranial neoplasia in horses is rare compared to other species. Detailed information such as neurological, electroencephalographic, and histopathological examination of horses with intracranial neoplasia associated with seizures is scarce in the literature. Furthermore, ganglioglioma has not been reported in the horse. A 7-year-old Quarter horse cross Paint gelding was examined due to recurrent seizure-like episodes of 1-year duration. The seizures had been increasing in frequency and length, occurring up to 20 times a day at the time of presentation. Neurological examination revealed intermittent obtundation and multiple left sided abnormalities consisting of upper motor facial and tongue hemiparesis, facial hyperesthesia and cervical hypoesthesia, proprioceptive deficits, thoracic limb hypermetria upon head elevation; and intermittent paroxysmal activity consistent with seizures. Cranial nerve reflexes were normal. Vocalization, conjugate vertical nystagmus, intermittent blindness, left sided head tilt and flexion of neck, and lack of response to environmental stimuli were observed during seizure activity. A right sided cerebrothalamic disease was suspected. An electroencephalogram confirmed seizure activity with main focus on the right side at the central, parietal, and occipital regions further supporting neuroanatomical localization. Additionally, subclinical paroxysmal activity was noted on the electroencephalogram. A ganglioglioma was identified in the right cerebrothalamic area, and other cranial parts of the brainstem based on immunohistochemical examination. To the authors' knowledge this is the first report of intracranial ganglioglioma in the horse. This intracranial neoplasia should be added to the possible causes of intracranial masses and seizures in horses.

## Background

Intracranial neoplasia in horses is rare. Furthermore, ganglioglioma has not been described in the horse. Gangliogliomas are slow-growing tumors which consist of mixed degeneration of glial and ganglion cells (1–3). These benign tumors of the central nervous system are rare in humans and animals with sporadic descriptions in dogs, rodents, a calf, and a European hedgehog (4–8). Gangliogliomas and gangliocytomas in humans constitute ~0.33–1.3% of primary central nervous system tumors (8). Gangliogliomas in children and young adults most frequently affect the temporal lobe followed by the frontal lobe. These tumors occasionally occur in the parietal and occipital lobes, less commonly in the brainstem, and rarely in the ventricles. Gangliogliomas are seldom outside the brain lobes (8). Epilepsy develops in 70–100% of affected patients (3, 8–11). Most gangliogliomas occur spontaneously in children, however, genetic syndromes such as neurofibromatosis have been associated as risk factors (12, 13). This report describes the clinical, electroencephalographic, and histopathological findings of a ganglioglioma in a 7-year old Quarter horse cross Paint gelding.

## Case Presentation

The present case report describes in detail the neurological and diagnostic examination of a horse presented for suspected seizures. The neuroanatomical localization of this horse's neurological deficits was the right cerebrothalamic region and caused by the presence of a ganglioglioma. This is the first report of ganglioglioma in the literature in horses.

The horse presented to the William R. Pritchard Veterinary Medical Teaching Hospital at the University of California, Davis with a history of repetitive seizures term used by the owner. These episodes of 1-year duration had been steadily increasing in frequency and length. Multiple veterinarians examined the horse and found mild osteoarthritis of the C5-6 vertebrae but no clinical or definitive diagnosis was reached for the primary complaint. The gelding was treated with flunixin meglumine at 1.1 mg/kg IV q12h (Merck Animal Health, Intervet Inc., Madison, New Jersey, USA) for 5 days with no improvement of signs. The abnormal episodes increased in frequency up to 20 times a day, occurring usually in clusters of 3–4 seizures in a 20-min time frame. The gelding was previously used for western pleasure and showmanship classes. At presentation the horse was receiving hydroxyzine hydrochloride at 1 mg/kg PO q12h (GlaxoSmithKline, Sacramento, California, USA) to manage recurrent urticaria.

**Figure 1:**
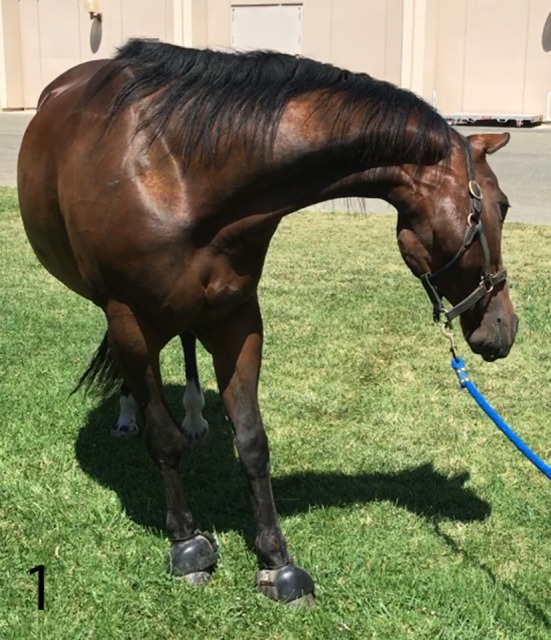
Horse while having seizures. Horse vocalized a high pitch whinny, appeared blind and unresponsive to the environment. Episodes lasted 5–10 s and occurred every 10 min.

The gelding was in good body condition (body condition score 5 of 9) and had normal physical and physiological parameters based on physical examination at our institution. Upon arrival, his mentation was bright and alert with intermittent periods of obtundation. As the examination progressed, his mentation became more consistently obtunded. Neurological examination revealed intermittent left sided facial hemiparesis (reduced facial muscle tone, droopy ear, ptosis, narrow nostril opening, muzzle slightly deviated to the right, reduced lip tone, and mild reduced prehension with food sticking out of the mouth on the left side), left sided facial hyperesthesia, reduced mastication on the left side, intermittent reduced tongue tone on the left side, and reduced cervical sensation on the left side. Other functions involving the facial nerve such as menace response and palpebral reflex were normal. Proprioception was reduced in all limbs but more pronounced on the left side. Dynamic examination revealed mild hypermetria of the left thoracic limb upon head elevation and no other gait abnormalities. During the examination, the horse had paroxysmal activity suggestive of seizures every 10 min. These episodes lasted 5–10 s each. During these episodes, the horse vocalized a high pitch whinny for 2–5 s, flared nostrils, tilted his head and flexed his neck tightly to the left toward his thorax, leaning (sometimes left or right), and developed a conjugate vertical nystagmus (Figure 1). Further, the horse appeared blind and unresponsive to the environment but remained standing. Initiation of paroxysmal activity frequently occurred after stimulation by a sudden noise or movement. In between paroxysmal activity, the horse performed chewing movements. Occasionally, the episodes would terminate with the horse performing a tight 360° spin to the right. The combination of left sided neurological abnormalities with intermittent spinning to the right, normal cranial nerves responses, reactions, and reflexes; supported a right sided supranuclear lesion resulting in contralateral upper motor neuron hemiparesis of mastication, facial and tongue function. Furthermore, intermittent paroxysmal activity suggestive of seizures was observed. The neuroanatomical localization was right sided cerebrothalamic disease.

A complete blood count and chemistry panel were within normal limits except for mildly elevated glucose (6.2 mmol/L [111 mg/dL], reference range 2.8–5.9 mmol/L [50–107 mg/dL]) and creatine kinase (446 IU/L, reference range 119–287 IU/L) concentrations and presumed to be secondary to transport or from recent seizure activity.

**Figure 2:**
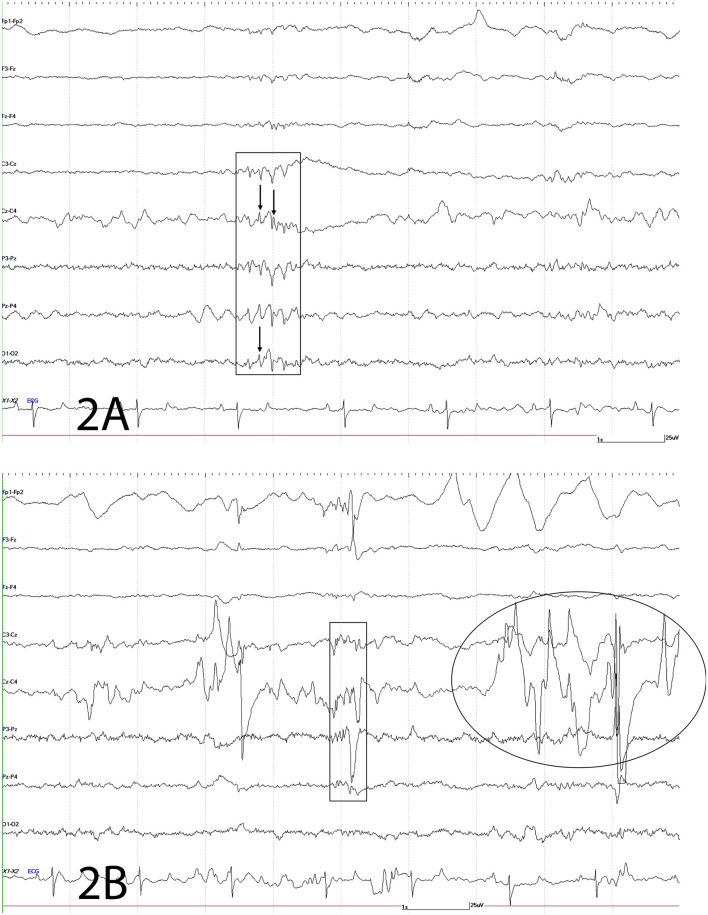
Electroencephalogram. The following figures depict EEG recordings at various stages. For all figures: odd numbers represent left side, even numbers represent right side, FP, frontal polar; F, frontal; C, central; P, parietal; O, occipital; z, midline; A, auricular; X2-X3 = ECG. Bar = 1 s, 25 mV. Note different scale for ECG. **(A)** Electroencephalogram. Rectangle depicts complex of spikes (thin arrow), sharps and waves at C3-Cz, Cz-C4, P3-Pz, Pz-P4, O1-O2; mainly right sided. **(B)** Electroencephalogram. This EEG depicts a combination of spikes, sharps, and waves (rectangle) and movement artifact intermittently shown from local movement [facial twitches (oval and large wave within rectangle)] as the result of paroxysmal activity.

An electroencephalogram (EEG) was performed to evaluate cerebral cortical activity and confirm paroxysmal activity consistent with seizures. Furthermore, EEG was also performed to detect the presence of subclinical seizures (14). Digital EEG with simultaneous video recording was performed using a Nihon Kohden digital wireless EEG 9000 system (Neurofax Wireless Input 1,000 A, Nihon Kohden America Inc., Foothill Ranch, California, USA). Electrode nomenclature and placement was based from a modified human 10–20 system and described in horses previously (15, 16). The horse was placed in a padded stall and sedated with detomidine hydrochloride at 0.01 mg/kg IV (Zoetis Inc., Orion Pharma, Kalamazoo, Michigan, USA) for electrodes placement. Needle electrodes were placed subcutaneously in the scalp for the EEG recording as follows: frontal-polar (2 electrodes), 3 frontal, 3 central, 3 parietal, and 2 in occipital regions. Additional electrodes included one that served as a ground (between the 2 frontal-polar electrodes) and one at the base of each ear to evaluate for movement artifacts on the EEG. Concurrently, an electrooculogram (two subcutaneous electrodes per eye, one each in the upper and lower eyelids), electromyogram (two subcutaneous electrodes located at the region of the splenius muscle), and electrocardiogram (one subcutaneous electrode in the region of the left heart base and one at the left heart apex) were also performed. A bipolar montage (rostral to caudal and transverse) was used with sensitivities set for recording as previously described (15, 17). Twenty minutes of EEG with simultaneous video recording were obtained for interpretation. Despite the lack of clinical manifestations, the EEG demonstrated paroxysmal activity consistent with seizures every 10–12 s (Figure 2) followed by facial/ear twitches every 1–5 min. Intermittent slowing of waves was observed in between paroxysmal activity (not shown). Toward the end of the recording, the horse initiated chewing movements similar to those observed prior to clinical manifestations of seizures. The paroxysmal activity consisted of sharp waves (70–200 ms), and spikes (<70 ms) and waves, and complexes of these supportive of seizure activity (Figure 2). Paroxysmal activity was observed on both sides but more prominent in the right side of the central, parietal, and occipital regions. The EEG findings further supported a right sided cerebrothalamic disorder.

Due to the profound clinical signs and poor prognosis, the owner elected euthanasia without further diagnostics. The horse was euthanized with intravenous concentrated pentobarbital sodium and phenytoin sodium at 100 mg/kg IV (Virbac AH, Inc., Fort Worth, Texas, USA), following sedation with xylazine hydrochloride at 1 mg/kg IV (Vet One, MWI, Boise, Idaho, USA) and submitted for post mortem evaluation. An atlanto-occipital cerebrospinal tap was performed within 5 min after euthanasia and submitted for cytology. The cerebrospinal fluid had a total protein of 82 mg/dL and total nucleated cell count of 3 cells/μL. The nucleated cells consisted of 87% small mononuclear (lymphocytes) and 13% large mononuclears with variably vacuolated macrophages consistent with mild mononuclear reactivity.

**Figure 3:**
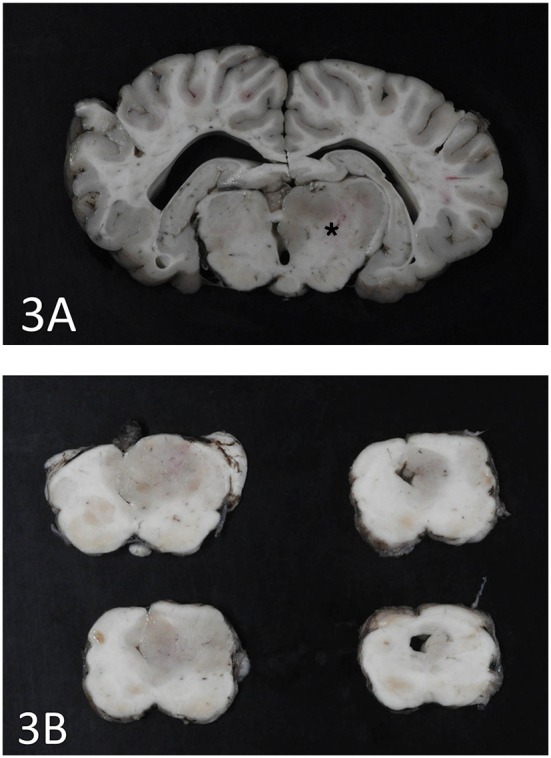
**(A)** Cerebrothalamus. Transverse section at the level of motor nuclei and hippocampus at the cerebrothalamus. Note discoloration and enlargement of the right side thalamic area (star). **(B)** Brainstem. Transverse sections at the level of the brainstem. The ganglioglioma is seen as a gray round area on the right side and partially occluding the mesencephalic aqueduct.

**Figure 4:**
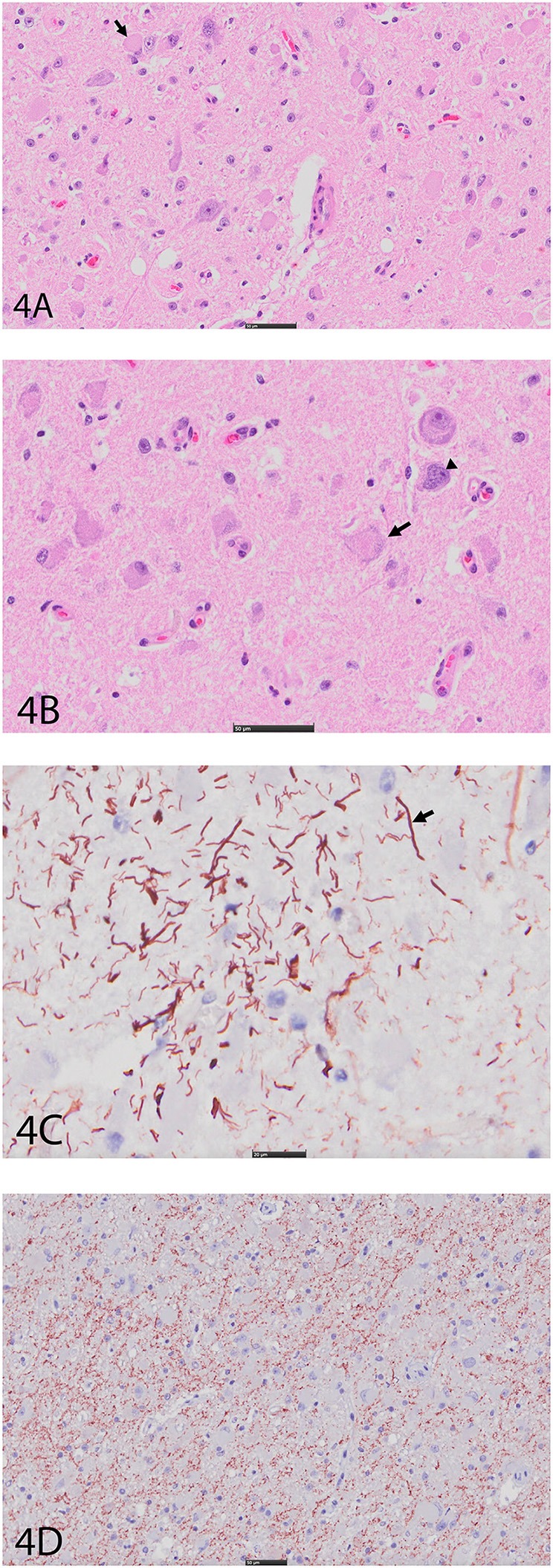
**(A)** Right cerebral cortex using hematoxylin and eosin (H&E). Note hypercellular region featuring large, pleomorphic neurons with vesicular (arrow), hyperchromatic nuclei, and indistinct, peripheralized Nissl substance. Smaller nuclei are also hypercellular and represent glial component. Bar = 50 μm. **(B)** Right cerebral cortex using H&E. Neurons often feature irregular profiles with coarse, peripheralized Nissl substance (arrow), and nuclear atypia (arrowhead). Bar = 50 μm. **(C)** Right cerebral cortex depicting GFAP immunohistochemistry. There is increased immunoreactivity within the mass, and cells feature thick, non-branching cytoplasmic processes (arrow) characteristic of neoplastic astrocytes. Bar = 20 μm. **(D)** Right cerebral cortex depicting synaptophysin immunohistochemistry. There is diffuse reactivity within the mass. Bar = 50 μm.

An expansile mass (3 × 2 cm) was present in the right side of the brain extending between the 3rd ventricle and geniculate nucleus at the level of the hippocampus (Figure 3A). The mass extended into the mesencephalon, partially compressing the mesencephalic aqueduct with no apparent ventriculomegaly (Figure 3B). The poorly defined mass consisted of neoplastic cells diffusely scattered within the neuropil of the affected region. The right piriform lobe and adjacent temporal lobe were mildly expanded, fluctuant, and more corrugated in texture than the left side structures. The lateral aspect of the right temporal lobe contained a poorly demarcated region (2 × 3 cm) of mottled pale pink to dark red discoloration. The left lateral temporal lobe contained a similar region of mottled pale pink to dark red discoloration measuring 1 × 0.5 cm. The geniculate nucleus was also involved. Immunohistochemistry revealed two populations of apparently neoplastic cells; one subset was immunoreactive to glial fibrillary acidic protein (GFAP) and the other was immunoreactive to synaptophysin (Figure 4). These results confirmed the tumor was composed of neoplastic glial and ganglion cells, findings most consistent with a ganglioglioma. A primary glial tumor was excluded due to the presence of atypical large cells with neuronal differentiation that were positive on immunohistochemistry for synaptophysin and negative for GFAP expression.

## Discussion

The horse in this report presented for an undiagnosed, protracted, progressive disease of 1-year duration. The horse had previously been treated for cervical osteoarthritis and presumption of having abnormal behavior or panic episodes. Seizure-like activity became more frequent and severe. The slow clinical progression and benign local expansion of ganglioglioma in this horse were similar to those described in humans. The tumor's predilection site of the temporal and frontal lobes commonly results in intractable seizures in humans (1, 2). Similar to human cases, this horse displayed seizures frequently with the EEG revealing subclinical seizure activity as frequently as every 10 s. Ventricular dilation and cleft formation in the globus pallidus secondary to increased cerebral pressure was described in a dog with a thalamic ganglioglioma (6). Hydrocephalus is a recognized sequela of human gangliogliomas that contributes to the progression of neurological signs (11, 13). The mass in this horse was impinging upon the mesencephalic aqueduct but no ventriculomegaly was noted. However, expansion of this mass would have likely resulted in ventriculomegaly and worsening of the neurological signs.

Gangliogliomas are extremely rare tumors of the central nervous system in humans and animals as evidenced by the scarce case reports (1–11). The majority of case reports in animals have encompassed an anatomical dichotomy of intracranial vs. extracranial (spinal cord) presentations, with a single case report in a dog of an unusual intraocular location (3–6). Gangliogliomas identified in the spinal cord of a calf and European hedgehog presented with ataxia, paraplegia and urinary bladder malfunction (3, 4).

Seizures are less commonly reported in horses compared to other species (18–22). Both, intracranial (e.g., congenital anomaly, granuloma, neoplasia, and abscess) and extracranial (e.g., metabolic such as hypoglycemia and hyperammonemia) causes of seizures have been documented in horses (23). Epilepsy is defined as a disease of the brain characterized by having two or more unprovoked seizures over 24 h apart. Epilepsy has been classified as primary or secondary. Primary includes confirmed or suspected genetic or familial origin commonly referred as idiopathic epilepsy. Secondary includes structural where epilepsy is provoked by intracranial or cerebral disease (e.g., vascular, inflammatory, infectious, trauma, anomaly, developmental, neoplasia, and degenerative). Reactive epilepsy is the reaction of the healthy brain to a transient systemic insult (e.g., hypoglycemia) (23). Similar attempts at classification of seizures/epilepsy have been made in horses (19). In this case, secondary epilepsy due to the presence of a mass occupying lesion compressing and deforming the brain mainly at the cerebrothalamic area resulted in chronic recurrent progressive seizures. Primary and secondary intracranial neoplasia in horses have been reported as both, extra- and intra-axial neoplasia. These include adenocarcinoma, astrocytoma, ependymoma, melanoma, meningioma, glioblastoma multiforme, and oligodendroglioma (24–30). Other non-infectious mass occupying lesions in horses include cholesterinic granuloma and pituitary adenoma (31).

## Concluding Remarks

This report describes in detail for the first time the clinical, laboratory, electroencephalographic, and immunohistochemical findings of a horse with ganglioglioma in the brain. This case also highlights the importance of early referral to a facility equipped with a board-certified neurologist, and diagnostic modalities for confirmation of seizures, diagnosis, and management if possible. This practice avoids unnecessary costs, early diagnosis and treatment, or early euthanasia if prognosis is poor due to cause, unmanageable seizures, and/or safety concerns. The location of the ganglioglioma in the brain of this horse and development of seizures as the most common clinical presentation are similar features to those reported in other species. However, motor facial dysfunction (with normal cranial nerve reflexes) as the result of a lesion in the cerebrothalamus (motor center in cerebral cortex and major motor relay center in the thalamus) was also a predominant feature in this horse (32). Intermittent blindness and vestibular signs (vertical nystagmus, head tilt, neck flexion, leaning) can also be explained by the tumor location as described in dogs with thalamic infarcts (33). These constellations of signs with concurrent EEG findings have not been reported in the horse. Furthermore, subclinical seizures can go undetected as evidenced in this horse unless an EEG is performed. Lastly, tumors of the central nervous system in the horse are rare.

## Ethics Statement

All aspects of patient examination, diagnostic investigation, and case management were within standard medical care. The owner consented the use of her horse's medical condition for publication and teaching purposes. No institutional animal care protocol was required.

## Author Contributions

CE-J and MA were the clinicians who examined the case, analyzed and interpreted the data, and wrote the manuscript. MA performed and interpreted neurological and electroencephalographic examination. KW, FM, and MR performed and described macroscopic and microscopic examination. All authors reviewed and approved the manuscript.

### Conflict of Interest

The authors declare that the research was conducted in the absence of any commercial or financial relationships that could be construed as a potential conflict of interest.

## Abbreviations

EEG: Electroencephalogram.
